# Commentary: Electron transport across the cell envelope via multiheme c-type cytochromes in *Geobacter sulfurreducens*

**DOI:** 10.3389/fchem.2025.1674350

**Published:** 2025-10-20

**Authors:** Derek R. Lovley

**Affiliations:** 1 Electrobiomaterials Institute, Key Laboratory for Anisotropy and Texture of Materials (Ministry of Education), Northeastern University, Shenyang, China; 2 Department of Microbiology, University of Massachusetts, Amherst, MA, United States

**Keywords:** electromicrobiology, microbial nanowires, e-pili, cytochrome nanowires, protein nanowires, extracellular electron transfer

## Introduction

In their review (Tabari and Hochbaum, 2025), Tabari and Hochbaum selectively cite a few references to make the claim that the predominant electrically conductive filaments emanating from *Geobacter sulfurreducens* are cytochrome filaments that are required for long-range extracellular electron transfer (EET). This commentary briefly outlines the extensive literature that Tabari and Hochbaum omitted that contradicts their claims.

### Filament identification

Tabari and Hochbaum assert that the extracellular filaments originally thought (Reguera et al., 2005) to be electrically conductive pili (e-pili) “are now widely accepted to be networks of MHCs [multi-heme *c*-type cytochromes]” and that “Evidence supporting this model and its reconciliation with previously published data have been extensively reviewed elsewhere.” However, all of the studies and reviews cited focus on cryo-electron microscopy. Preparing samples for cryo-electron microscopy requires many processing steps that can specifically enrich for cytochrome filaments (Lovley and Walker, 2019). Neither Tabari and Hochbaum nor the reviews they cite acknowledge published alternative experimental approaches specifically designed to avoid processing artifacts that demonstrate that there are abundant e-pili as well as cytochrome filaments emanating from cells.

**FIGURE 1:**
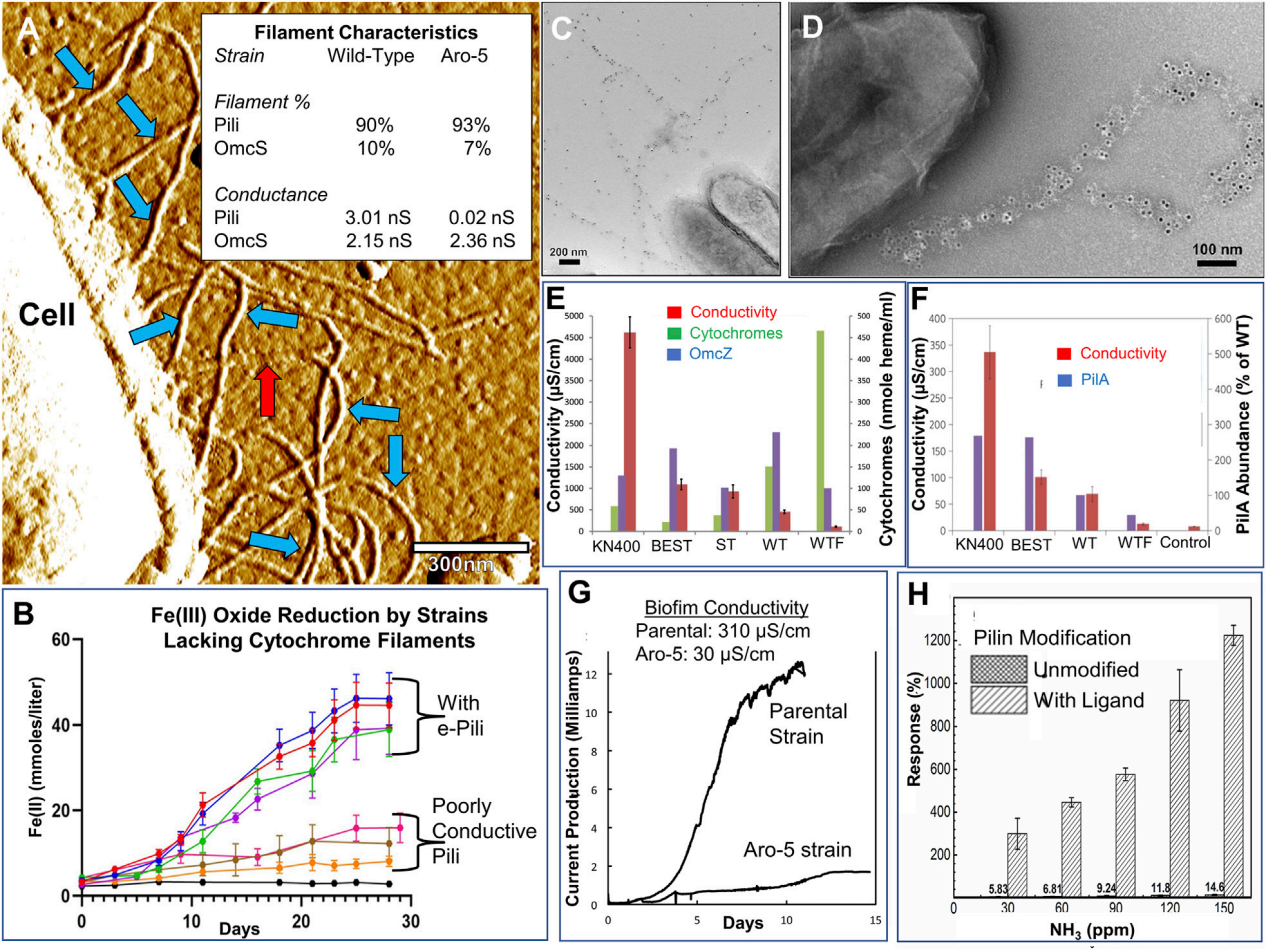
Examples of data demonstrating the assembly of the *G. sulfurreducens* pilin into e-pili, the greater abundance of e-pili compared to cytochrome filaments in unprocessed cells, the important role of e-pili in EET, and that cytochrome filaments are not required for Fe(III) oxide reduction and are not the primary contributors to biofilm conductivity. **(A)** Atomic force microscopy image of filaments emanating from a hydrated, unfixed cell that was drop cast directly onto a conductive surface. Blue arrows designate filaments with a diameter, morphology, and conductance comparable to the filaments that *E. coli* produces when expressing the *G. sulfurreducens* pilin gene. Red arrows designate filaments with the 4 nm diameter and unique longitudinal morphology of OmcS filaments (Image modified from Liu et al., 2021). Inset table: abundance and conductance data for the wild-type strain and strain Aro-5, in which the wild-type pilin gene was replaced with a pilin gene modified to yield pili with lower conductivity (Data from Liu et al., 2021). **(B)** Data (Schwarz et al., 2024) demonstrating that strains in which the genes for one, two, or all three of the filament-forming cytochromes were deleted effectively reduced Fe(III) oxide to Fe(II). In contrast, Fe(III) oxide reduction is impaired in strains expressing a gene that yield poorly conductive pili. **(C,D)** Transmission electron micrographs (Ueki et al., 2019) of immunogold labelling of cells demonstrating that when a pilin gene that encodes a his-tag at the carboxyl end of the pilin is introduced the filaments emanating from cells contain the his-tag, providing further evidence that the filaments are comprised of pilin. **(E)** Data from current-producing biofilms (Malvankar et al., 2012) demonstrating that deletion of genes for the filament-forming cytochromes OmcS and OmcE increased biofilm conductivity and that there was no correlation between the abundance of OmcZ and biofilm conductivity. Abbreviations: KN400, strain of *G. sulfurreducens* selected for superior current production; BEST, strain in which the genes for OmcB, OmcE, OmcS, and OmcT were deleted; ST, strain in which the genes for OmcS and OmcT were deleted; WT, wild-type strain; WTF, wild-type strain grown on electrodes, but with fumarate as the electron acceptor. **(F)** Data (Malvankar et al., 2011) comparing the conductivity of biofilms of various strains and demonstrating that a greater abundance of the pilin protein, PilA, is associated with higher biofilm conductivity. Abbreviations the same as in panel **(E)**. Control designates system with no cells. **(G)** Data (Vargas et al., 2013) demonstrating that substituting a pilin gene that yields pili with lower conductivity results in a strain, Aro-5, that produces much less current than the parental strain and that the conductivity of the Aro-5 biofilms are greatly reduced compared to the parental strain. **(H)** Data (Lekbach et al., 2023) demonstrating that modifying the *G. sulfurreducens* pilin gene heterologously expressed in *E. coli* to contain a short carboxyl peptide designed to bind ammonia substantially increases the electronic response of purified nanowires harvested from *E. coli*. This response further demonstrates that *E. coli* assembles the *G. sulfurreducens* pilin into e-pili. All images reproduced with permission.

For example, e-pili, not cytochrome filaments predominate when *G. sulfurreducens* is examined with methods that avoid sample pretreatment (Liu et al., 2021; Liu et al., 2022; Schwarz et al., 2024) (see Figure 1A for an example). In those studies, cells were grown with fumarate as the electron acceptor, one of the same growth conditions in what Tabari and Hochbaum claim was the “landmark study…demonstrating that the conductive extracellular nanowires are, in fact, composed of polymerized OmcS.” A small drop of live culture was directly drop-cast onto a flat conductive surface (Liu et al., 2021; Liu et al., 2022; Schwarz et al., 2024). There was no fixation. The cells remained hydrated while they were examined with high resolution atomic force microscopy (AFM).

AFM revealed (Figure 1A) that ca. 10% of filaments emanating from wild-type *G. sulfurreducens* had the 4 nm diameter and unique longitudinal morphology characteristic of OmcS filaments (Liu et al., 2021). Conductive AFM tip analysis demonstrated conductance through the filaments to the underlying surface, consistent with the proposed conductivity of OmcS filaments. The remaining ca. 90% of the filaments (Figure 1A) had the same 3 nm diameter, morphology, and conductance (Liu et al., 2021) as the filaments that *E. coli* produces when heterologously expressing the *G. sulfurreducens* pilin gene, PilA (Ueki et al., 2020). These 3 nm diameter conductive filaments were the only filaments observed when the genes of all three of the known filament-forming cytochromes were deleted (Schwarz et al., 2024). Thus, when processing artifacts were avoided, cytochrome filaments were a minor component of the overall *G. sulfurreducens* nanowire complement. The vast majority of the nanowires had the same properties as e-pili.

When the wild-type pilin gene was replaced with a modified pilin gene known to yield less conductive pili when expressed in *E. coli* (Sonawane et al., 2025), ca. 90% of the filaments had the same 3 nm diameter as in the wild-type cells, but the conductance was orders of magnitude lower (Liu et al., 2021). As in the parental strain, OmcS filaments accounted for the other 10% of the filaments and their conductance was unchanged. These results are consistent with the expectation that changing the amino acid composition of the pilin would change the conductance of the pili, but not the conductance of OmcS or other filaments not comprised of pilin.

Furthermore, when the PilA gene expressed in *G. sulfurreducens* was modified to encode short peptide sequences at the carboxyl end of the pilin protein, the predominant filaments emanating from the cells displayed those peptides (Ueki et al., 2019) (see Figures 1C,D for examples). The expression of 3 nm diameter conductive filaments in *E. coli* and other microbes, as well as the ability to engineer dramatic changes in their conductivity and binding properties through targeted modifications of the pilin amino acid sequence (see Figure 1H for an example), further demonstrates that the *G. sulfurreducens* pilin assembles into conductive filaments (Adhikari et al., 2016; Lekbach et al., 2023; Liu et al., 2019; Sonawane et al., 2025; Szmuc et al., 2023; Tan et al., 2017; Ueki et al., 2019). Although 6.5 nm diameter filaments containing PilA were described in some mutant strains, as previously reviewed in detail, they have never been observed in wild-type strains and are a mutation artifact (Lovley, 2022).

### Filament function

Tabari and Hochbaum critique early studies on e-pili function (Reguera et al., 2005), but ignore later work showing that replacing the wild-type pilin gene with variants yielding poorly conductive pili addressed concerns about cytochrome mislocalization (Liu et al., 2014; Liu et al., 2021; Liu et al., 2022; Schwarz et al., 2024; Steidl et al., 2016; Ueki et al., 2018; Vargas et al., 2013). Expressing poorly conductive pili impairs EET even when cytochromes are properly localized (see Figures 1B,G for examples).

Tabari and Hochbaum claim that “cleaner background strains with all cytochrome nanowires deleted for genetic studies” are needed, failing to recognize that such a strain already exists (Schwarz et al., 2024). It effectively reduced Fe(III) oxide in the absence of all three filament-forming cytochromes. Conductive e-pili were still required (Schwarz et al., 2024).

Tabari and Hochbaum state that “Immunolabeling indicates that OmcZ is found widely dispersed within the conductive matrix of *G. sulfurreducens* biofilms.” The study cited (Inoue et al., 2010) actually reported the opposite finding: “OmcZ was highly concentrated at the biofilm–electrode interface.” This specific localization suggests that OmcZ has a specialized role in electron transfer to the electrode, not long-range electron transport through the bulk conductive biofilm, a conclusion further supported by the lack of correlation between OmcZ abundance and biofilm conductivity (Malvankar et al., 2012). Deleting genes for OmcS and OmcE not only did not inhibit current production, it increased biofilm conductivity (Malvankar et al., 2012) (Figure 1E). In contrast, there is a positive correlation between pilin abundance and biofilm conductivity (Malvankar et al., 2011) (Figure 1F) and expressing poorly conductive pili substantially lowers biofilm conductivity and current production (Vargas et al., 2013) (Figure 1G).

## Conclusion

There is a substantial body of literature that supports the role of e-pili in *G. sulfurreducens* EET (Guberman-Pfeffer et al., 2024; Lovley, 2022; Lovley and Holmes, 2022; Schwarz et al., 2024). Cytochrome filaments are not essential for Fe(III) oxide reduction. Their role in other forms of EET requires further clarification.
